# The Impact of Minimally Invasive Surgical Modality and Task Complexity on Cognitive Workload: An fNIRS Study

**DOI:** 10.3390/brainsci15040387

**Published:** 2025-04-08

**Authors:** Fuat Ücrak, Kurtulus Izzetoglu, Mert Deniz Polat, Ümit Gür, Turan Şahin, Serhat Ilgaz Yöner, Neslihan Gökmen İnan, Mehmet Emin Aksoy, Cengizhan Öztürk

**Affiliations:** 1 The Institute of Biomedical Engineering, Boğaziçi University, Istanbul 34342, Turkey; fuat.ucrak@std.bogazici.edu.tr (F.Ü.); cozturk@bogazici.edu.tr (C.Ö.); 2 School of Biomedical Engineering, Science and Health Systems, Drexel University, Philadelphia, PA 19104, USA; mp3677@drexel.edu; 3 General Surgery, Istanbul Haseki Training and Research Hospital, Istanbul 34250, Turkey; umit_gur@yahoo.com; 4 Gynecologic Surgery, Sancaktepe Sehit Prof. Dr. Ilhan Varank Training and Research Hospital, Istanbul 34785, Turkey; dr.turanshn@gmail.com; 5 Department of Biomedical Device Technology, Acibadem Mehmet Ali Aydinlar University, Istanbul 34752, Turkey; serhat.yoner@acibadem.edu.tr (S.I.Y.); emin.aksoy@acibadem.edu.tr (M.E.A.); 6 Department of Computer Engineering, Koç University College of Engineering, Istanbul 34450, Turkey; ninan@ku.edu.tr

**Keywords:** minimally invasive surgery, laparoscopic surgery, robotic surgery, cognitive workload, functional near-infrared spectroscopy (fNIRS), surgical training

## Abstract

Background: Minimally invasive surgical techniques, including laparoscopic and robotic surgery, have profoundly impacted surgical practice by improving precision, reducing recovery times, and minimizing complications. However, these modalities differ in their cognitive demands and skill acquisition requirements, which can influence the learning curve and operative performance. To assess and evaluate this variability across these modalities, a functional near-infrared spectroscopy (fNIRS) system is used as an objective method for monitoring cognitive activity in surgical trainees. fNIRS can provide insights and further our understanding of the mental demands of different surgical techniques and their association with varying task complexity. Objective: This study seeks to assess the influence of surgical modality (laparoscopy vs. robotic surgery) and task complexity (pick and place (PP) vs. knot tying (KT)) on cognitive workload through fNIRS. We compare real-world and simulation-based training environments to determine changes in brain activation patterns and task performance. Methods: A total of twenty-six surgical trainees (general and gynecologic surgery residents and specialists) participated in this study. Participants completed standardized laparoscopic and robotic surgical tasks at varying levels of complexity while their cognitive workload was measured using fNIRS. This study included both simulation-based training and real-world surgical environments. Hemodynamic responses in the prefrontal cortex (PFC), task completion times, and performance metrics were analyzed. Results: Laparoscopic surgery elicited higher activity changes in the prefrontal cortex, indicating increased cognitive demand compared with robotic surgery, particularly for complex tasks like knot tying. Task complexity significantly influenced mental load, with more intricate procedures eliciting greater neural activation. Real-world training resulted in higher cognitive engagement than simulation, emphasizing the gap between simulated and actual surgical performance. Conclusions: Cognitive workload was lower and significantly different during robotic surgery than during laparoscopy, potentially due to its ergonomic advantages and enhanced motor control. Simulation-based training effectively prepares surgeons, but the cognitive workload results indicate that it may not fully replicate real-world surgical environments. These findings reveal the importance of cognitive workload assessment in surgical education and suggest that incorporating neuroimaging techniques such as fNIRS into training programs could enhance skill acquisition and performance.

## 1. Introduction

Minimally invasive surgical techniques such as laparoscopy and robotic surgery have transformed patient care by offering smaller incisions and greater operative precision. Over the past three decades, laparoscopic surgery has rapidly advanced, with significant improvements in instrumentation, visualization, and surgical techniques, establishing it as a standard of care across numerous surgical specialties. Despite its more recent emergence, robotic surgery has proven equally transformative, revolutionizing the field by providing surgeons with enhanced dexterity, precision, and three-dimensional visualization [1]. Robotic-assisted surgery has gained widespread adoption in various surgical specialties, particularly urology, gynecology, and general surgery. While robotic platforms reduce tactile feedback compared with laparoscopy, they compensate through enhanced 3D visualization and intuitive controls. Studies have shown that improved depth perception reduces reliance on spatial memory and facilitates faster decision-making, thereby affecting cognitive resource allocation [2,3]. Additionally, laparoscopic and robotic surgery often result in shorter hospital stays, faster recovery times, and less postoperative pain compared with traditional open surgery [1,2,3,4].

For patients, these techniques reduce surgical trauma and recovery time. These advantages often lead to improved patient outcomes, enhanced quality of life, and a faster return to normal activities [2,3,5].

While both laparoscopic and robotic surgery training aim to develop minimally invasive surgical skills, they differ in terms of technology, learning curves, and training tools. Laparoscopic training is more widely accessible and forms the foundation for robotic surgery, while robotic surgery training focuses on mastering advanced instrumentation and console operation [1,5,6,7]. Both require dedicated practice and ongoing skill maintenance. Although both are minimally invasive, laparoscopy and robotic surgery differ in ergonomics, instrument control, and learning curves [2,3,6,8]. Effective surgical training must address not only psychomotor skills but also the cognitive demands associated with complex procedures.

Over the last three decades, simulation has become a cornerstone of medical education, providing a risk-free environment for acquiring technical skills, promoting patient safety, and mitigating medical errors, thus enhancing physician training and improving healthcare outcomes [9,10,11]. To ensure patient safety during complex laparoscopic and robotic surgical operations, surgeons must undergo rigorous training with advanced simulation modalities that replicate real-world scenarios and challenges. Training curricula such as the Fundamentals of Laparoscopic Surgery (FLS) and Fundamentals of Robotic Surgery (FRS) integrate simulation and structured skill assessments to ensure safe operative performance. Training protocols like Fundamentals of Laparoscopic Surgery (FLS) and Fundamentals of Robotic Surgery (FRS) are widely used to train surgeons for these surgical modalities [12,13].

The Fundamentals of Laparoscopic Surgery (FLS) program is a comprehensive curriculum designed to teach and assess the basic skills required for laparoscopic surgery. It includes online modules covering cognitive knowledge, hands-on skills training on simulators, and a standardized assessment to measure proficiency. The FLS program is widely used to train surgeons, assess competency, and ultimately improve patient safety by ensuring surgeons possess the necessary skills for laparoscopic procedures [14].

Like the FLS program for laparoscopy, the FRS curriculum uses simulation and virtual reality to train robotic skills such as console operation and 3D navigation [15].

While simulation improves technical proficiency, it does not fully capture the mental workload experienced during actual procedures. Neuroimaging tools like fNIRS offer an objective physiological approach to assess this cognitive dimension of surgical performance. Beyond traditional performance metrics, brain-based measures obtained through functional neuroimaging techniques can provide deeper insights into trainee’s performance [16]. Various neuroimaging tools have been employed to measure the underlying neural mechanisms involved in cognitive processes. Neuroimaging techniques such as functional magnetic resonance imaging (fMRI), electroencephalography (EEG), magnetoencephalography (MEG), positron emission tomography (PET), and functional near-infrared spectroscopy (fNIRS) offer alternative approaches for performance assessment [17]. Given its noninvasive, wearable, and user-friendly nature, the fNIRS system offers significant advantages for measuring prefrontal cortex activity in field settings. Consequently, fNIRS has been selected as the neuroimaging technique for this study. Characterized by its lightweight design, low cost, ease of setup, and relative robustness to movement artifacts, fNIRS offers a promising approach for brain imaging studies [17,18,19,20,21,22,23,24,25]. Several studies have successfully utilized brain imaging data acquired by fNIRS systems in conjunction with existing scoring systems during simulation-based medical training [17,18,19,21,22,25,26,27].

Functional near-infrared spectroscopy (fNIRS) is a neuroimaging technique that leverages the optical properties of biological tissues and hemoglobin chromophores. fNIRS employs wavelengths ranging from 700 to 900 nm, within which most biological tissues, including neural tissues, are transparent. The primary absorbers at these wavelengths are the chromophores of oxygenated and deoxygenated hemoglobin (HbO and HbR). By analyzing the transmission of light through cortical tissue using the modified Beer–Lambert Law, the concentrations of oxygenated and deoxygenated hemoglobin can be calculated. Changes in these hemoglobin concentrations are directly linked to variations in brain activity [17,18,22,23]. The prefrontal cortex (PFC), the anterior region of the frontal lobes, plays a crucial role in a range of higher-order cognitive functions, including attentional control, emotional regulation, complex learning, and social cognition, and is implicated in executive functions, behavioral inhibition, and general intelligence [28,29,30,31]. The dorsolateral prefrontal cortex (DLPFC) is a key component of the prefrontal cortex, which is the brain region primarily responsible for executive functions such as planning, strategy building, and executive decision-making [32]. The anterior medial prefrontal cortex (AMPFC) is a key region involved in a wide range of higher-order cognitive functions, including task management, planning, reasoning, attention, multitasking, task set representations, and decision-making [33,34]. Neuroimaging studies consistently demonstrate that PFC exhibits increased activity during tasks with high cognitive demands, suggesting its crucial role in executive functions such as working memory and decision-making [25,35,36].

This research investigates brain activation, specifically hemodynamic responses in attention and working memory areas, during both simulated and real-world laparoscopic and robotic surgery training, with the goal of comparing the collected data across these two settings.

The hypotheses of this study are as follows:

- For complex surgical tasks, mental demand would be different between laparoscopic and robotic surgery, and this difference can be determined by identifying cognitive workload using neurophysiological measures from the prefrontal cortex.
- Regardless of surgical modality (laparoscopic or robotic), increasing task complexity (e.g., knot tying vs. PP) will result in higher task load and thus elevated cognitive workload.

## 2. Materials and Methods

### 2.1. Participants

A group of volunteers participated in this study, including n = 21 resident medical doctors from the General and Gynecologic Surgery departments, along with 5 specialists in General Surgery and Gynecologic Surgery. A total of n = 26 participants, comprising 14 males and 12 females, were included in the study. The participants had an average age of 30 ± 3 years, with a median age of 29 years (range: 26 to 35 years). All participants were right-handed, except for one male who was left-handed. Although all participants were novices in robotic surgery, they had comparable laparoscopic surgery experience. The details of the participants are provided in Table 1. One participant, due to time constraints, was unable to fully participate in the study and was therefore excluded. Exclusion of this participant did not significantly affect the demographic balance or statistical power of the analysis. This study was approved by the Ethical Committee of Acibadem Mehmet Ali Aydinlar University (Registration number: ATADEK-2023/05/164) and conducted in accordance with the Declaration of Helsinki, with all participants providing written informed consent.

### 2.2. Experimental Protocol

To objectively quantify participants’ cognitive workload in real-time during simulated laparoscopic and robotic surgical procedures, functional near-infrared spectroscopy (fNIRS) sensors were employed. Two standardized surgical tasks were used for this study: knot tying (KT), a complex task, and pick and place (PP), a simpler dexterity-based task. These task types are referred to as KT and PP in the rest of the manuscript. Laparoscopic and robotic surgery tasks with different difficulty levels were chosen for simulation sessions. After a baseline measurement with fNIRS, participants first completed a low-difficulty pick and place (PP) task for familiarization. Task difficulty was calibrated based on the complexity of motor coordination and time to completion, with knot tying (KT) designated as the complex task. Participants then completed both PP and KT tasks using laparoscopic and robotic systems.

To ensure comprehensive data collection and analysis, all simulated laparoscopic and robotic surgery tasks were recorded on video and observed by trained medical specialists enabling precise calculation of the time taken to complete each task. This comprehensive approach allowed for a detailed examination of participant performance, task complexity, and the impact of workload and stress on surgical outcomes.

KT and PP are fundamental skills in laparoscopic surgery, providing a solid foundation for more complex procedures [35]. These tasks are crucial for developing the fine motor skills, hand–eye coordination, and spatial awareness necessary for successful laparoscopic surgery [36].

PP is another vital skill that helps trainees develop dexterity and control within the confined space of the laparoscopic field [35]. This task involves manipulating objects with forceps or other instruments, requiring precision and coordination [35,37]. PP exercises can improve a surgeon’s ability to handle delicate tissues, suture needles, and other instruments without causing damage.

Both KT and PP are essential for developing the fundamental skills required for laparoscopic surgery [38]. They provide a solid foundation for more advanced procedures and help to reduce the risk of complications. As such, these tasks are commonly included in laparoscopic surgery training programs [38]. In this study, the term ‘real-world’ refers to task performance on physical box trainers or robotic systems in simulated operating room conditions, rather than live patient surgery.

#### 2.2.1. Laparoscopic Surgery Training Protocol

**Figure 1:**
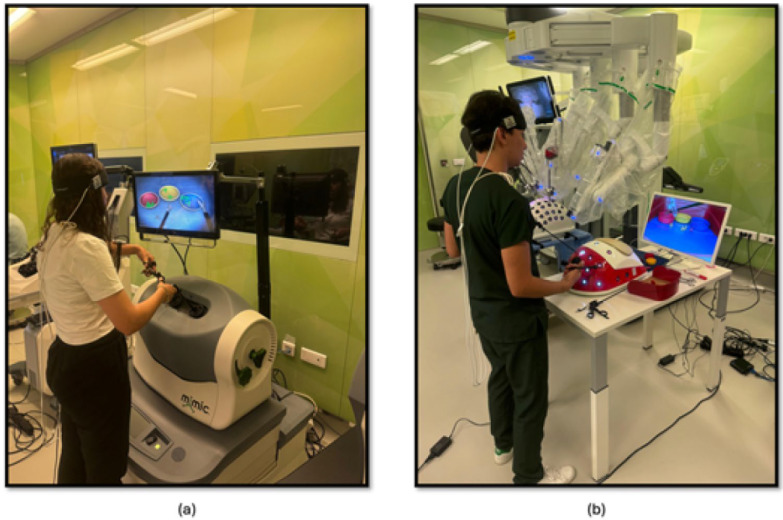
(**a**) PP module on the laparoscopic surgery simulator; (**b**) PP module on the laparoscopic surgery box trainer.

**Figure 2:**
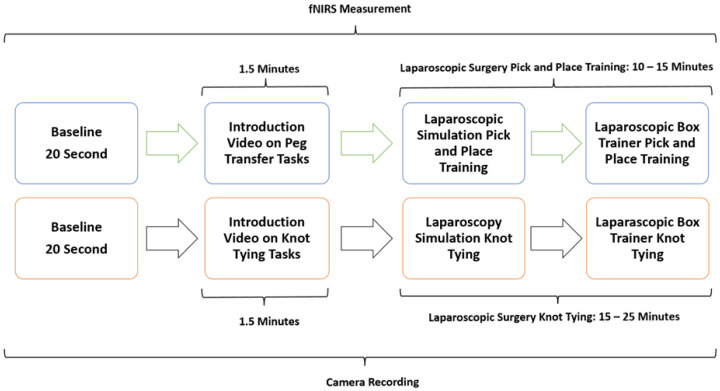
Workflow of PP and KT using both a laparoscopic surgery simulator and a real-life laparoscopic box trainer.

The study methodology involved a four-stage process laparoscopic simulation and box training with real laparoscopic instrumentation. Initially, participants underwent familiarization training with both the laparoscopic simulator and box trainer to ensure familiarization with the equipment. Subsequently, baseline measurements were collected using fNIRS to establish a baseline for physiological responses. Following this, participants engaged in PP (Figure 1a) using the laparoscopic simulator (the Xperience Team Trainer™ (Mimic Technologies, Seattle, WA, USA), focusing on mastering the fundamental movements and techniques required for laparoscopic surgery. Finally, participants transitioned to the laparoscopic box trainer (Figure 1b), replicating the PP in a more realistic environment. This sequential approach allowed for a systematic evaluation of the effectiveness of both simulation and box training in developing laparoscopic skills and understanding the associated physiological responses. The workflow of the first part of the study is shown in Figure 2.

**Figure 3:**
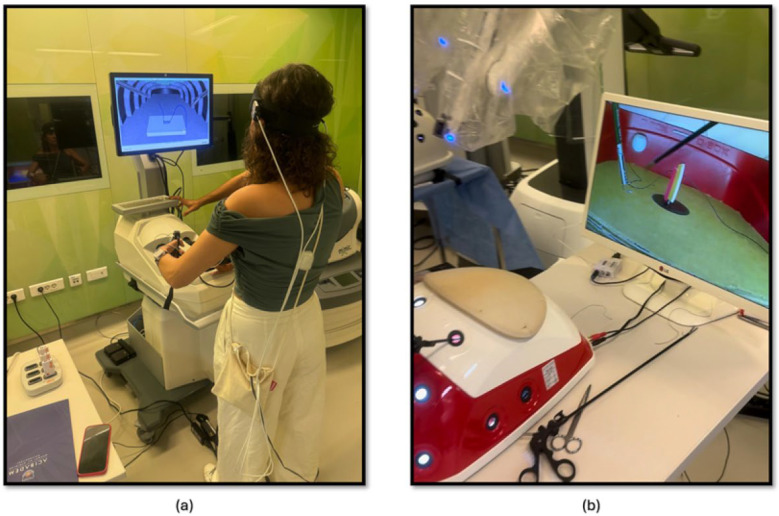
(**a**) KT module on the laparoscopic surgery simulator; (**b**) KT module on the laparoscopic surgery box trainer.

The second part of this study focused on comparing the effectiveness of laparoscopic surgery simulation and box trainer training in improving KT skills. Participants underwent baseline measurements using fNIRS to assess their physiological responses during cognitive load. Subsequently, they received training in KT (Figure 3a) using both laparoscopic surgery simulation (LapVR simulator, CAE Healthcare, Saint-Laurent, QC, Canada) and box trainer platforms. The training sessions were designed to familiarize participants with the equipment, techniques, and challenges associated with laparoscopic KT. Following the training, participants performed KT (Figure 3b) using both platforms, allowing for a direct comparison of their performance and physiological responses. The workflow of the second part of the study is shown in Figure 2.

#### 2.2.2. Robotic Surgery Training Protocol

**Figure 4:**
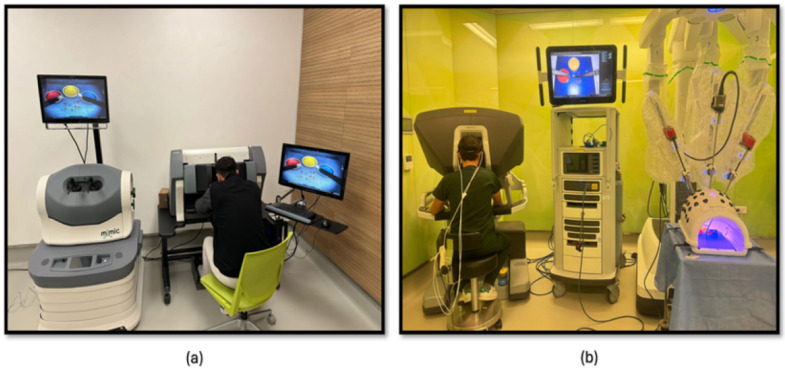
(**a**) PP module on the robotic surgery simulator; (**b**) PP training module on the robotic surgery platform.

**Figure 5:**
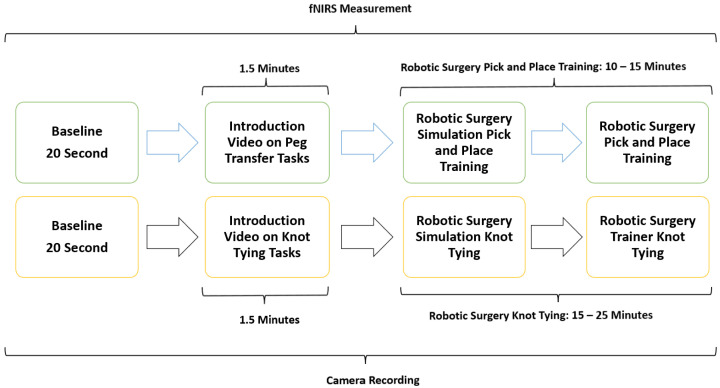
The workflow of the study included PP and KT using both a robotic surgery simulator and a real-life robotic surgery platform.

The third phase of the study involved PP using both a robotic simulator and a real robotic surgery robot. This training was designed to familiarize participants with the intricacies of robotic surgery and to assess their performance and physiological responses. Participants were first trained on the robotic surgery simulator (Figure 4a), Mimic^®^ dV-Trainer (Mimic Technologies, Seattle, WA, USA), to develop fundamental skills and to become accustomed to the robotic interface. Subsequently, they transitioned to the real robotic surgery robot (Figure 4b), da Vinci Surgical System (Intuitive Surgical, Sunnyvale, CA, USA), where they performed the same PP in a simulated surgical environment. This sequential approach allowed for gradual progression in complexity and ensured that participants were adequately prepared before engaging in the actual robotic system. The workflow of the third part of the study is shown in Figure 5.

**Figure 6:**
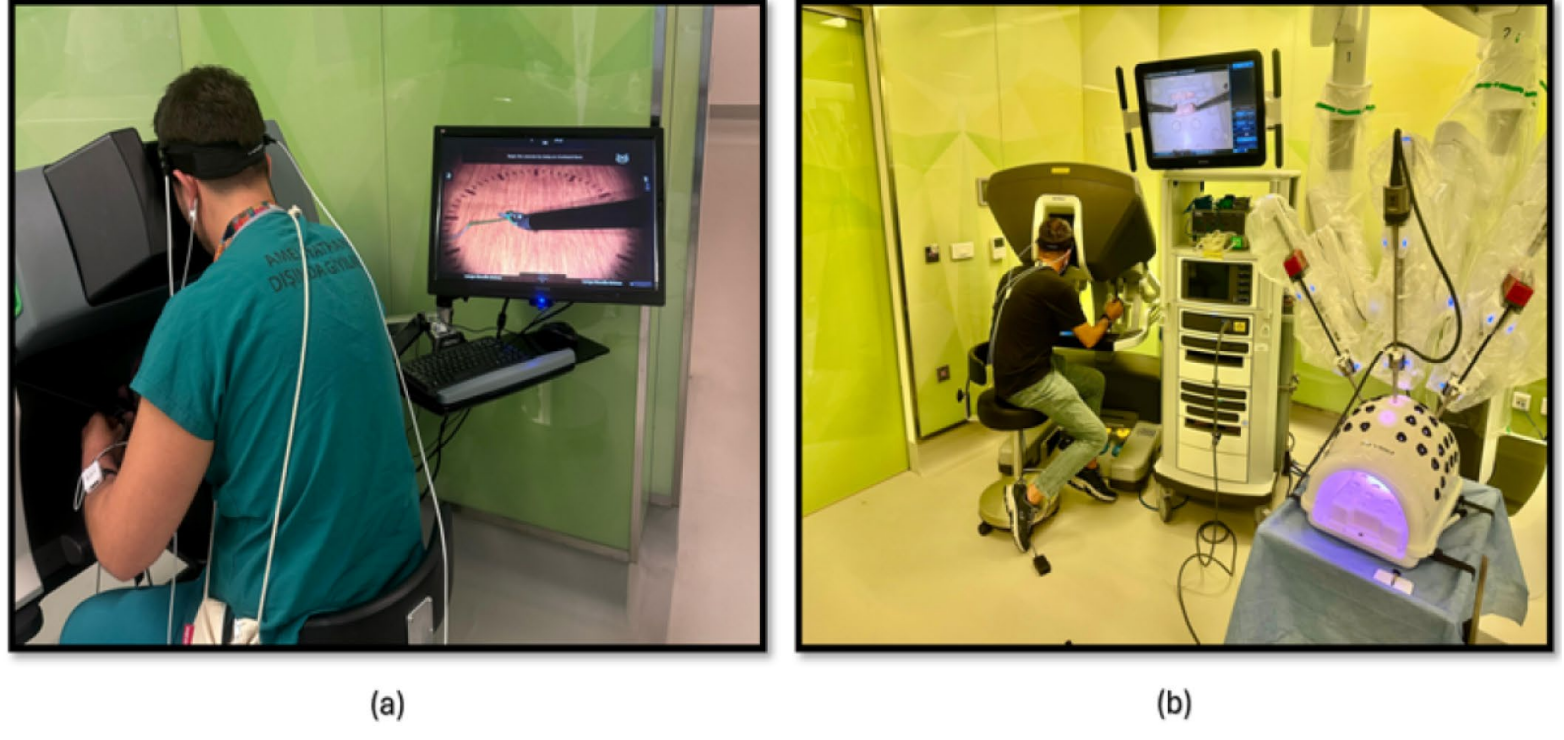
(**a**) KT module on the robotic surgery simulator; (**b**) KT training module on the robotic surgery platform.

The fourth phase of the study involved conducting KT training sessions using both the robotic simulator (Figure 6a) and the robotic surgery robot (Figure 6b). Participants were initially trained on the robotic simulator to familiarize themselves with the system’s controls, haptic feedback, and visual interface. Once comfortable with the simulator, they transitioned to the robotic surgery robot for hands-on KT practice. Throughout the training sessions, physiological data, fNIRS, were continuously measured to assess participants’ cognitive load, and brain activation patterns during the task. The workflow of the fourth part of the study is shown in Figure 5.

### 2.3. fNIRS Recording and Preprocessing

**Figure 7:**
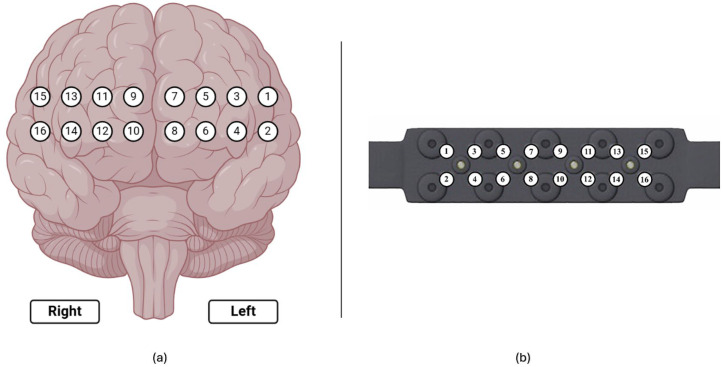
(**a**) Corresponding 16 optodes locations over the prefrontal cortex; (**b**) fNIRS probe with 4 light sources and 10 detectors.

To monitor hemodynamic responses in the prefrontal cortex (PFC), participants were equipped with a continuous wave functional near-infrared spectroscopy (fNIRS) device (fNIR Devices, LLC, Potomac, MD, USA). This device utilized an 18-channel probe positioned over the PFC (Figure 7a), comprising 4 LED sources emitting light at 730 nm and 850 nm wavelengths and 12 photodetectors including 2 short-separation channels as shown in Figure 7b). To ensure accurate time synchronization during post-processing, the start and end times of each session and simulation task were marked and time synchronized.

The quality of fNIRS signals can be compromised by multiple noise sources, such as instrument noise (e.g., light source instability, electronic noise), physiological interference (e.g., respiration, heartbeat), and motion artifacts, necessitating effective noise removal as a preliminary step in data processing [39,40,41].

Head movements, causing relative shifts between source–detector pairs and the scalp, can introduce motion artifacts into fNIRS signals, manifesting as rapid, large-magnitude spikes that significantly exceed tissue-related hemodynamic changes [42,43]. Motion artifacts, a significant source of noise in fNIRS experiments, were addressed using a wavelet-based filter. Channel rejection and baseline normalization ensured cleaner signals for analysis. Physiological signals like heart rate (over 0.5 Hz) and respiration (over 0.2 Hz) exhibit higher frequency ranges than hemodynamic responses and instrument-degradation-induced noise (3–5 Hz) [39,41]. To isolate the lower-frequency hemodynamic signals, a linear phase low-pass FIR filter with a cutoff frequency of 0.1 Hz was applied to the raw light intensity signals.

Following artifact removal, filtering, and channel rejection procedures, for each participant, changes in oxy-Hb and deoxy-Hb concentrations (µmol/L) over time were calculated using the modified Beer–Lambert Law based on optical density (OD) changes measured at 730 and 850 nm [39,40]. This allowed for the quantitative estimation of key hemodynamic parameters on a channel-specific basis. These parameters included oxy-hemoglobin (HbO) and deoxy-hemoglobin (HbR).

A 10-s baseline measurement [23,40] was acquired from each participant after a 20-s relaxation period, prior to laparoscopic and robotic surgery training. Changes in HbO and HbR concentrations were calculated relative to this baseline throughout the duration of each task.

An 18-channel fNIRS system, sampling at 5 Hz, was employed, targeting the prefrontal cortex (PFC) with a sensor configuration comprising 4 LEDs and 10 detectors. This system comprised 16 long-separation channels for cortical activity measurements.

### 2.4. Statistical Analysis

Little’s Missing Completely at Random (MCAR) test was conducted to assess if missing data were randomly distributed. The results indicate that the missing data were MCAR (χ^2^ = 5.163, *p* = 1.000, df = 441), allowing for unbiased parameter estimation in subsequent analyses. Given the presence of missing data and the repeated measures structure, a linear mixed-effects regression (LME) approach was employed to analyze the dependent variables: Completion Time and Mean ΔHbO. Mean ΔHbO represents the average change in oxygenated hemoglobin from baseline, a validated neurophysiological index of cognitive effort. LME models were used to account for both fixed effects (Session, Task Complexity, Modality, Environment) and random effects (individual participants). Each participant was modeled with a random intercept to control for baseline inter-individual differences. It is an important neurophysiological marker of cerebral oxygenation and neural activation, particularly in cognitive workload assessment, skill acquisition analysis, and task efficiency evaluation.

Since surgery requires high cognitive load, motor coordination, and decision-making, tracking Mean ΔHbO in different brain regions can help understand how trainees develop expertise and manage cognitive resources. Before model fitting, missing values were checked and imputed when necessary, and outliers were removed using the Z-score method within each session separately to avoid excessive data exclusion. Additionally, log10 transformation was applied to dependent variables when normality assumptions were violated.

To account for within-subject dependencies, two linear mixed-effects (LME) models were tested. Each participant was modeled with a random intercept to control for baseline inter-individual differences, and fixed effects were included for Session, Modality, Environment, and Task Complexity. The first model included ‘Session’ as a fixed effect, where Session was a categorical variable representing the combination of task repetitions and simulation type. The second model treated Modality (Laparoscopic vs. Robotic), Media (Real vs. Simulation), and Scenario (KT vs. PP) as separate fixed effects to assess their individual contributions. In both models, participants were modeled as random intercepts to account for inter-individual variability in task performance.

Model selection was performed using maximum likelihood estimation (ML) to allow for valid model comparisons based on Akaike Information Criterion (AIC) and Bayesian Information Criterion (BIC). ML is appropriate when comparing nested models. AIC evaluates goodness-of-fit while penalizing model complexity. BIC is similar to AIC but applies a stricter penalty for complexity. Since some models initially failed to converge, the Powell optimization method was used as an alternative, as it is particularly effective for complex, non-convex problems. Additionally, robust standard errors were applied where heteroscedasticity was detected, ensuring more reliable parameter estimates. To determine the best-fitting model, comparisons were made based on AIC, BIC, log-likelihood, deviance, marginal R^2^ (variance explained by fixed effects), conditional R^2^ (variance explained by both fixed and random effects), and the intraclass correlation coefficient (ICC) to estimate the proportion of variance attributable to participant-level differences. Model assumptions, including normality and homoscedasticity of residuals, were assessed through Shapiro–Wilk tests, Levene’s test, and residual diagnostic plots. Multicollinearity was evaluated using the Variance Inflation Factor (VIF), and robust standard errors were applied in cases where homogeneity of variance assumptions were violated.

For significant fixed effects, Tukey’s Honest Significant Difference (HSD) test was used for post hoc pairwise comparisons. Multiple comparisons in post hoc analyses were controlled using the Benjamini–Hochberg False Discovery Rate (FDR) method. Effect sizes for contrasts were reported using Cohen’s d. While no continuous covariates were included in the current models, the LME framework supports their inclusion.

Cohen’s d was computed to determine effect sizes, where d = 0.2 was considered a small effect, d = 0.5 a medium effect, and d = 0.8+ a large effect [44].

All statistical analyses were performed in Python (version 3.13) using the statsmodels package [45] for mixed-effects modeling, pinguin for missing data analysis and effect size calculations [46], scipy for statistical testing [47], and matplotlib and seaborn [48] for visualization and assumption checking.

## 3. Results

In the following analyses, two dependent variables were modeled: (i) Completion Time in seconds (log-transformed for normality) and (ii) Mean ΔHbO, defined as the average change in oxygenated hemoglobin (HbO) concentration from baseline, expressed in micromolar (µM). These variables were modeled using the linear mixed-effects models explained earlier.

### 3.1. Results for Completion Time

As participants performed surgical tasks under different conditions, log-transformed completion time was used as the dependent variable to ensure model assumptions were met. Two model structures were tested. The Session model treated each unique combination of Modality (Laparoscopic vs. Robotic), Environment (Simulation vs. Real), and Task Type (KT vs. PP) as a single categorical variable. This approach captures performance differences across discrete task conditions. The Full Factorial model, by contrast, included Modality, Environment, and Task Type as independent fixed effects, assuming additive and interaction effects. Model fit comparisons between these structures are shown in Table 2. The model with ‘Session’ as a fixed effect outperformed the full factorial model (Modality + Environment + Task Complexity) in explaining variance in completion time, as indicated by a lower AIC (279.589 vs. 307.826) and BIC (312.216 vs. 327.402) values. The likelihood ratio test confirmed a significant effect of ‘Session’ compared with the interaction model (χ^2^ = 36.232, *p* < 0.001), indicating that treating Session as a single categorical variable better captured performance differences. The intraclass correlation coefficient (ICC) was 0.120, indicating that participant-level variance accounted for a small portion of the total variability in completion time (Table 2).

**Figure 8:**
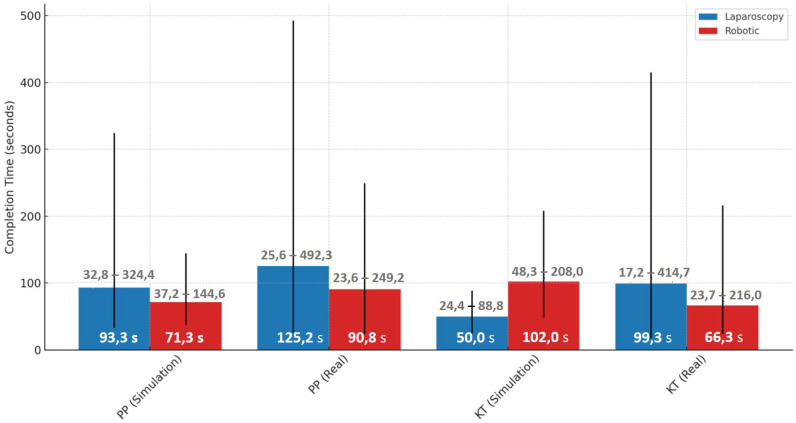
Median completion times with min-max ranges for laparoscopic and robotic tasks in simulation and real environments.

Performance differences were evident when comparing simulated and real environments across both laparoscopic and robotic modalities. For the laparoscopy PP task, the median completion time increased substantially from 93.3 s [85.0–102.6] in simulation to 125.2 s [113.4–143.7] in the real environment. Similarly, for the laparoscopy KT task, the median time rose from 49.0 s [45.2–70.6] in simulation to 99.2 s [89.4–115.0] in the real setting. In the robotic PP task, the median time increased from 71.3 s [61.2–83.6] in simulation to 90.8 s [84.2–104.8] in real operations. A different trend was seen in the robotic KT task, where median completion time dropped from 101.9 s [90.6–126.4] in simulation to 66.3 s [60.3–74.1] in the real setting, suggesting differences in task complexity or familiarity across environments (Figure 8).

**Figure 9:**
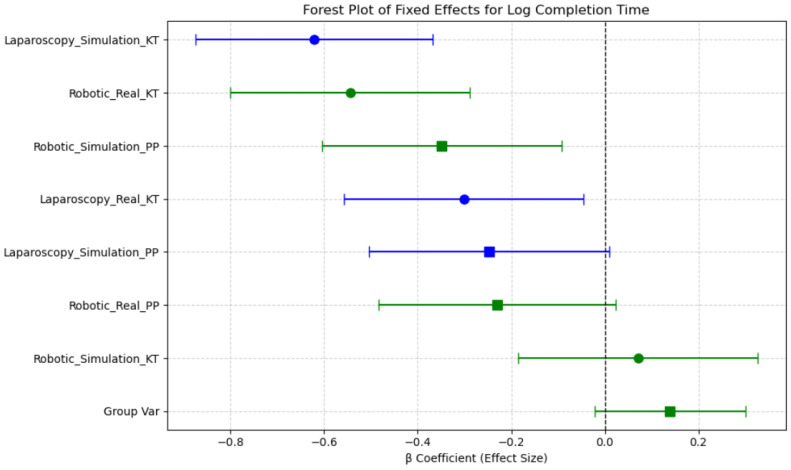
Forest plot of fixed effects for log completion time across sessions.

β coefficients represent estimated differences in log(Completion Time)/Mean ΔHbO relative to a reference session, derived from the linear mixed-effects model. The estimated fixed effects (β coefficients) and their 95% confidence intervals (CIs) indicate which sessions were associated with increased or decreased completion times compared with the reference category. The intercept (β = 4.19, *p* < 0.001) represents the baseline log-transformed completion time, indicating the expected task duration when all other factors are at their reference levels. Sessions such as Robotic Simulation PP and Robotic Real PP were associated with reduced completion times, as evidenced by their negative β coefficients, while Laparoscopy Simulation KT and Robotic Simulation KT exhibited increased completion times. However, confidence intervals overlapping zero suggest that not all session effects reached statistical significance. Notably, the confidence intervals for Robotic Simulation KT and Laparoscopy Simulation KT remain above zero, suggesting these conditions significantly increase completion time relative to the reference. In contrast, the Robotic Real KT session demonstrated a reduction in completion time, indicating improved efficiency in real-world conditions compared with simulated environments (Figure 9).

These findings support the role of session-based learning effects, where task duration varies based on the combination of Modality, Environment, and Task Complexity. The observed reductions in completion time for robotic real tasks suggest a potential transfer of skills from simulated training to real-world execution, highlighting the importance of structured training protocols.

Overall, completion times were shorter in real conditions compared with simulations, for robotic tasks, as seen in the significant difference between Robotic Simulation KT and Laparoscopy Simulation KT (*p* < 0.001, d = −2.342). These results indicate that task modality (robotic vs. laparoscopy), simulation environment, and task repetition significantly influence completion time, with robotic tasks requiring longer durations than laparoscopic tasks in general. The transition from simulation to real environments improves efficiency, particularly for robotic tasks (Table 3).

These results suggest that simulation-based training significantly influences task duration, particularly in robotic environments, where task completion time improves with real-world exposure. These findings suggest that Session-based task grouping better captures meaningful differences in task performance than analyzing Modality, Environment, and Task Complexity independently. Additionally, the observed learning effects in simulated conditions highlight the impact of task repetition on skill acquisition.

### 3.2. Results for Mean ΔHbO

As participants performed surgical tasks under different conditions, Mean ΔHbO was used as the dependent variable to assess cortical hemodynamic responses across task conditions. The model with ‘Session’ as a fixed effect outperformed the full factorial model (Modality + Environment + Task Complexity) in explaining variance in Mean ΔHbO, as indicated by a lower AIC (842.519 vs. 882.962) and BIC (875.093 vs. 902.507) values. The likelihood ratio test confirmed a significant effect of ‘Session’ compared with the interaction model (χ^2^ = 48.444, *p* < 0.001), indicating that treating Session as a single categorical variable better captured neural activity differences. The intraclass correlation coefficient (ICC) was 0.144, indicating that participant-level variance accounted for a moderate proportion of total variability in Mean ΔHbO (Table 2).

A clear neural adaptation, i.e., training effect was observed across repeated tasks in both laparoscopic and robotic simulations. Specifically, in the laparoscopy simulation, Mean ΔHbO significantly decreased from 3.252 [1.594–4.867] µM in the first task (KT) to 0.557 [−0.337–0.928] µM in the second task (PP), suggesting reduced cognitive load over time as participants become more familiar with tasks. Similarly, in the robotic simulation, Mean ΔHbO decreased from −1.600 [−2.861–−0.088] µM in KT to −0.069 [−0.651–1.293] µM in PP, indicating improved neural efficiency with task repetition.

**Figure 10:**
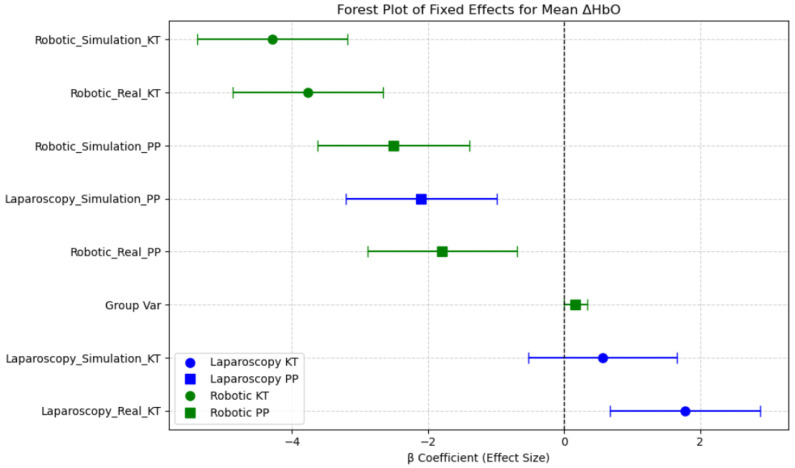
Forest plot of fixed effects for Mean ΔHbO across sessions.

The estimated fixed effects (β coefficients) and their 95% confidence intervals (CIs) illustrate which session conditions were associated with increased or decreased neural activation in the prefrontal cortex. The intercept (β = 4.46, *p* < 0.001) represents the baseline oxygenated hemoglobin level, indicating the expected Mean ΔHbO when all other factors are at their reference levels. Sessions such as Robotic Simulation KT, Robotic Real KT, and Laparoscopy Simulation PP showed significantly lower Mean ΔHbO levels, with the β coefficients well below zero and confidence intervals not overlapping zero, suggesting reduced prefrontal cortex activation in these conditions. In contrast, Laparoscopy Real PP and Laparoscopy Simulation KT demonstrated relatively higher Mean ΔHbO levels compared with robotic tasks, indicating greater mental demand. This aligns with the expectation that laparoscopic procedures require higher cognitive load and motor coordination compared with robotic-assisted surgeries, which offer more ergonomic control. Notably, Robotic Simulation PP had the largest negative effect on Mean ΔHbO, suggesting minimal neural activation in this session. The reduction in prefrontal cortex activity across robotic conditions could be indicative of increased automation in robotic systems, reducing the cognitive burden on surgeons. These findings emphasize the disparity between neural efficiency and cognitive workload across surgical modalities and training environments (Figure 10).

Overall, this analysis highlights the impact of task type, modality, and environment on neural activation patterns in the PFC region, underlining the role of session-based learning and task familiarity in surgical performance and training.

Post hoc pairwise comparisons revealed several significant differences in Mean ΔHbO across sessions (Table 3). Robotic tasks exhibited significantly lower Mean ΔHbO compared with laparoscopic tasks, particularly in robotic simulations. The largest reduction was observed in Robotic Simulation KT compared with Laparoscopy Real KT (*p* < 0.001, d = 2.269), indicating a substantial difference in hemodynamic responses between robotic and laparoscopic environments. Training in a simulated laparoscopic environment significantly reduced Mean ΔHbO. Specifically, Laparoscopy Simulation PP had significantly lower ΔHbO than Laparoscopy Real KT (*p* < 0.001, d = 1.953), suggesting higher adaptation to task in the simulation setting. Real robotic tasks resulted in lower Mean ΔHbO compared with simulated robotic tasks. Robotic Real KT had significantly lower Mean ΔHbO than Robotic Simulation KT (*p* < 0.001, d = 1.862), indicating increased neural efficiency in real robotic environments. Task complexity played a role in hemodynamic response, as Laparoscopy Simulation PP had lower Mean ΔHbO than Laparoscopy Simulation KT (*p* = 0.001, d = 1.413), suggesting an adaptation and training effect with repeated exposure. Between-modality comparisons showed that laparoscopic real tasks elicited significantly greater Mean ΔHbO compared with robotic real tasks. Laparoscopy Real KT had higher Mean ΔHbO than Robotic Real KT (*p* < 0.001, d = 2.235), indicating higher cognitive and physiological demands during laparoscopic procedures.

Overall, these results suggest that task modality (robotic vs. laparoscopy), simulation environment, and task repetition significantly influenced changes in the Mean ΔHbO, with robotic simulations showing the lowest activation in the PFC region, and real laparoscopic tasks requiring higher cognitive effort.

The findings further emphasize that Session-based task grouping better captures meaningful differences in cortical activity compared with independent analysis of Modality, Environment, and Task Complexity. Additionally, the observed neural adaptation effects in simulated conditions highlight the impact of task repetition on cognitive workload and skill acquisition.

## 4. Discussion

This study investigates behavioral and neural activity changes in laparoscopic and robotic surgery training, with a focus on the modality effect, task complexity effect, and experience level. Additionally, the comparison of simulation-based training and real-life systems provided insights into skill acquisition and neural adaptation to select tasks. Furthermore, the learning effect was analyzed by evaluating changes in completion time and neural activity between simulation and real-world tasks.

### 4.1. Modality Effect

The results demonstrate that surgical modality significantly influenced task performance and cognitive load. These outcomes align with prior fNIRS and EEG-based studies showing greater prefrontal activation during tasks requiring high executive control and motor precision [23,49,50]. Our findings contribute additional evidence supporting the value of portable neuroimaging for real-time assessment in surgical education. Laparoscopic surgery imposed a higher cognitive demand compared with robotic surgery for the Pick-and-Place task, as indicated by prefrontal cortex oxygenation levels. Real-world training induces significantly higher neural activity indicating increased cognitive workload and executive function demand. However, no significant differences in cognitive workload (HbO changes) or task performance (completion time) were observed between simulation-based and real-world training for the Pick-and-Place task in robotic surgery. These results suggest that for a basic motor task like Pick-and-Place, modality may not strongly impact cognitive engagement in robotic surgery training.

On the other hand, when a more complex task, such as knot-tying, was used in the study, modality effects led to noticeable changes. Laparoscopy exhibited higher cortical activation (HbO levels, suggesting increased cognitive and attentional demands. The higher HbR levels in laparoscopy further indicate greater oxygen consumption and task-related neuronal activity. These findings suggest that laparoscopy requires greater cognitive engagement and task-related brain activity, whereas robotic procedures, while taking longer to complete, may demand less cognitive effort. Robotic platforms appear to ease cognitive load through ergonomic design and simplified control interfaces. These results reinforce the idea that robotic systems reduce prefrontal cortical activation during complex tasks, likely due to ergonomic advantages, tremor filtration, and improved visualization, thereby reducing the need for compensatory cognitive effort [51].

### 4.2. Task Complexity Effect

Task complexity significantly influenced performance, as observed in the differences between knot tying (KT) and pick and place transfer (PP) tasks. Mean ΔHbO levels indicated a higher cognitive load in laparoscopic real tasks compared with robotic tasks, supporting previous findings that laparoscopic procedures require higher cognitive and motor coordination efforts [3]. The higher cognitive demand observed in laparoscopic real PP tasks posits the necessity for enhanced training protocols tailored to task complexity. Cognitive workload was lower and significantly different during robotic surgery than during laparoscopy, potentially due to its ergonomic advantages and enhanced motor control. Additionally, differences in cognitive load may also stem from task familiarity, as all participants had prior experience with laparoscopic surgery but were novices in robotic procedures [52,53].

### 4.3. Simulation vs. Real-Life Systems

A key finding of this study is the different impact of simulation versus real-life surgical environments on both performance metrics. Robotic simulations showed the highest cortical activation levels, indicating greater cognitive demand. The largest reduction in Mean ΔHbO was observed in robotic simulation KT compared with laparoscopic real KT (*p* < 0.001, d = 2.269), indicating a substantial difference in hemodynamic responses between robotic and laparoscopic environments. Conversely, real-life robotic surgery conditions facilitated improved efficiency, as seen in reduced completion times and lower Mean ΔHbO levels. This suggests that while simulation-based training is essential for initial skill acquisition, real-world exposure is crucial for refining sensorimotor skills and reducing cognitive load. While simulation-based training is essential for skill acquisition, the difficulty in transferring these skills to real-world applications underscores the need for enhanced training strategies. Our findings are consistent with prior studies showing reduced prefrontal cortex activation in robotic modalities [3], highlighting reduced cognitive load and ergonomic benefits. These results suggest incorporating fNIRS-based cognitive monitoring in training protocols may help tailor training intensity and modality for individual learners. Integrating tools like fNIRS into training programs may help tailor instruction based on cognitive demand. For instance, monitoring trainee workload could be used to adapt task difficulty in real time, identify cognitive overload, and promote personalized learning pathways, leading to more effective and safer surgical training.

## 5. Conclusions

Robotic surgery imposes a lower cognitive load than laparoscopy, contributing to better performance consistency across different training modalities. Moreover, complex tasks require higher cognitive and motor demands. The learning effect was evident in the prolonged completion times and increased neural activity in real-world settings, especially for laparoscopic surgery. While simulation-based training is essential for skill acquisition, the difficulty in transferring these skills to real-world applications underscores the need for enhanced training strategies. Robotic surgery demonstrated a more seamless transition, supporting its potential to reduce cognitive effort and improve skill retention.

Assessing cognitive skills by using real-time neuromonitoring may have the potential to provide a more effective way to differentiate levels of robotic or laparoscopic surgical expertise in parallel to tool-based metrics. Future training programs should integrate neurocognitive monitoring tools such as fNIRS to dynamically tailor the intensity and content of surgical instruction based on real-time cognitive workload. This personalized approach may accelerate learning, prevent cognitive overload, and improve long-term retention.

Future research should expand upon these findings by incorporating a larger sample size, including experienced Da Vinci robot surgeons, to enhance the generalizability of the results. Although our current modeling approach prioritizes interpretability and robustness in a small-sample context, future work may explore deep learning-based transfer learning frameworks.

## 6. Limitations

This study faced two primary limitations. First, the participating doctors’ busy clinical schedules created time constraints, limiting the exploration of alternative study designs and more in-depth investigation. Second, due to their demanding schedules, recruiting a larger number of surgical experts proved difficult. A total of 26 surgical trainees (general and gynecologic surgery residents and specialists) participated, providing a range of laparoscopic experience. However, only five were considered experts (all of whom were novices in robotic surgery) highlighting a key constraint in participant expertise. Additional potential limitations, such as learning effects from participants performing multiple tasks, should also be acknowledged. Future research could address these limitations by expanding participant diversity, incorporating more advanced simulation technologies, and adopting study designs that enable more comprehensive investigations.

## Figures and Tables

**Table 1:** Subject demographics.

Subject	Age	Gender	Handedness	Specialization (in Surgery)	Experience Level (Year)
Resident (R)					
R1	30	Male	Right	General	3.5
R2	28	Male	Right	General	1.0
R3	29	Female	Right	General	3.0
R4	28	Female	Right	General	2.0
R5	27	Male	Right	General	1.5
R6	33	Male	Right	General	2.0
R7	28	Female	Right	Gynecology	0.5
R8	28	Female	Right	Gynecology	0.5
R9	29	Female	Right	Gynecology	1
R10	26	Female	Right	Gynecology	0
R11	29	Male	Right	Gynecology	0
R12	35	Male	Right	Gynecology	0
R13	28	Female	Right	Gynecology	0.5
R14	33	Female	Right	Gynecology	0
R15	28	Female	Right	Gynecology	0
R16	26	Male	Right	Gynecology	0
R17	26	Male	Right	Gynecology	0.5
R18	28	Female	Right	Gynecology	2
R19	34	Male	Right	Gynecology	1
R20	31	Male	Right	Gynecology	2
R21	31	Male	Right	Gynecology	3
Expert (E)					
E1	35	Male	Left	Gynecology	3.0
E2	34	Female	Right	Gynecology	3.5
E3	34	Female	Right	Gynecology	4
E4	32	Male	Right	Gynecology	4
E5	33	Male	Right	Gynecology	4

**Table 2:** Model performance comparisons between the Session-based model and the Full Factorial model.

Dependent Variable	Log10 Transform of Completion Time logLik		Mean ΔHbO	
Main Effect	Session	Full Factorial *	Session	Full Factorial *
AIC	279.589	307.826	842.519	882.962
BIC	312.216	327.402	875.093	902.507
Log Likelihood	129.794	−147.913	−411.259	−435.481
Deviance	259.589	295.826	822.519	870.962
R^2^ Conditional	0.305	0.094	0.491	0.351
R^2^ marginal	1.000	1.000	0.564	0.416
ICC	0.120	0.074	0.144	0.099

* Modality + Environment + Task Complexity.

**Table 3:** The post hoc comparisons for ΔHbO and the log10 transform of completion time.

Contrast		Completion Time				Mean ΔHbO		
		Mean Diff. Log10	Mean Diff. Seconds	Adj. *p* *	Cohen’s d	Mean Diff.	Adj. *p* *	Cohen’s d
Lap_Real_KT	Lap_Real_PP	0.299	1.99	0.417	−0.436	−1.808	0.077	0.791
Lap_Real_KT	Lap_Sim_KT	−0.318	0.48	0.322	0.558	−1.248	0.468	0.523
Lap_Real_KT	Lap_Sim_PP	0.050	1.12	1.000	−0.082	−3.943	<0.001	1.953
Lap_Real_KT	Rob_Real_KT	−0.219	0.6	0.795	0.374	−5.520	<0.001	2.235
Lap_Real_KT	Rob_Real_PP	0.074	1.19	1.000	−0.107	−3.607	<0.001	1.791
Lap_Real_KT	Rob_Sim_KT	0.378	2.39	0.142	−0.680	−6.100	<0.001	2.269
Lap_Real_KT	Rob_Sim_PP	−0.034	0.92	1.000	0.060	−4.275	<0.001	1.989
Lap_Real_PP	Lap_Sim_KT	−0.617	0.24	0.001	1.238	0.560	0.985	−0.256
Lap_Real_PP	Lap_Sim _PP	−0.249	0.56	0.654	0.456	−2.134	0.017	1.195
Lap_Real_PP	Rob_Real_KT	−0.518	0.3	0.010	1.006	−3.712	<0.001	1.626
Lap_Real_PP	Rob_Real_PP	−0.225	0.6	0.749	0.359	−1.799	0.075	1.011
Lap_Real_PP	Rob_Sim_KT	0.079	1.2	0.999	−0.164	−4.292	<0.001	1.705
Lap_Real_PP	Rob_Sim_PP	−0.333	0.46	0.278	0.680	−2.467	0.003	1.277
Lap_Sim_KT	Lap_Sim_PP	0.368	2.33	0.157	−0.942	−2.695	0.001	1.413
Lap_Sim_KT	Rob_Real_KT	0.099	1.26	0.997	−0.287	−4.272	<0.001	1.795
Lap_Sim_KT	Rob_Real_PP	0.392	2.47	0.098	−0.785	−2.359	0.004	1.240
Lap_Sim_KT	Rob_Sim_KT	0.696	4.97	<0.001	−2.342	−4.852	<0.001	1.862
Lap_Sim_KT	Rob_Sim_PP	0.284	1.92	0.471	−0.924	−3.028	<0.001	1.480
Lap_Sim_PP	Rob_Real_KT	−0.269	0.54	0.573	0.653	−1.577	0.199	0.783
Lap_Sim_PP	Rob_Real_PP	0.024	1.06	1.000	−0.043	0.336	0.999	−0.237
Lap_Sim_PP	Rob_Sim_KT	0.328	2.13	0.295	−0.885	−2.157	0.015	0.948
Lap_Sim_PP	Rob_Sim_PP	−0.084	0.82	0.999	0.221	−0.333	1.000	0.207
Rob_Real_KT	Rob_Real_PP	0.292	1.96	0.448	−0.567	1.913	0.049	−0.952
Rob_Real_KT	Rob_Sim_KT	0.597	3.95	0.001	−1.844	−0.580	0.984	0.216
Rob_Real_KT	Rob_Sim_PP	0.185	1.53	0.903	−0.555	1.244	0.514	−0.580
Rob_Real_PP	Rob_Sim_KT	0.304	2.01	0.379	−0.630	−2.493	0.002	1.097
Rob_Real_PP	Rob_Sim_PP	−0.108	0.78	0.995	0.219	−0.669	0.962	0.418
Rob_Sim_KT	Rob_Sim_PP	−0.412	0.39	0.079	1.465	1.824	0.079	−0.762

* *p*-values were adjusted with false discovery rate (FDR).

## Data Availability

The data presented in this study are available on request from the corresponding author due to privacy concerns regarding participant information.

## Abbreviations

The following abbreviations are used in this manuscript:

fNIRS	Functional Near-Infrared Spectroscopy
FLS	Fundamentals of Laparoscopic Surgery
FRS	Fundamentals of Robotic Surgery
Hb	Hemoglobin
HbO2	Oxygenated hemoglobin
HbR	Deoxygenated hemoglobin
PFC	Prefrontal Cortex
LAMPFC	Left Anterior Medial Prefrontal Cortex
LDLPFC	Left Dorsolateral Prefrontal Cortex
RAMPFC	Right Anterior Medial Prefrontal Cortex
RDLPFC	Right Dorsolateral Prefrontal Cortex
KT	Knot tying
PP	Pick and place
RNI	Relative neural involvement
RNE	Relative neural efficiency
ROI	Region of interest
